# Supplementary material for: Remating responses are consistent with male postcopulatory manipulation but not reinforcement in D. pseudoobscura
Source: Ecol Evol. 2016 Dec 20;7(2):507–15. doi: 10.1002/ece3.2628 (PMC5243186; doi:10.1002/ece3.2628)
Supplement: Supplementary file 2 [file ECE3-7-507-s002.docx]

Supporting Information: Jeremy S. Davis, Dean M. Castillo, and Leonie C. Moyle “Remating responses are shaped by male post-copulatory manipulation but not reinforcement in *D.*

*pseudoobscura”*

# Supporting Methods

## Mating and Remating assay

The mating procedure was as follows:

1. Day 0: Virgin *D. pseudoobscura* females for each isofemale line were collected within 1 day of eclosion and maintained in groups of 5-10 for 5 days prior to mating, to ensure they reached sexual maturity and were receptive to first matings. Virgin males for all 5 populations/species identities were also collected on the same day. Males were isolated individually to increase receptivity to mating prior to introduction of females (Noor 1997).
2. Day 1: Virgin female *D. pseudoobscura* were transferred individually to vials with a single isolated male, without anesthesia. Assignment of females to each male type was at random. Each pair was observed for 3 hours upon transfer, and copulation latency (time to initiating copulation) and duration (length of copulation) were recorded. Regardless of whether a mating was observed within the first 3 hours, pairs were maintained together for 24 hours.
3. Day 2: Males were removed from Day 1 mating vials. Females were left in the vial to lay eggs.
4. Day 5: Virgin male flies from each *D. pseudoobscura* population were isolated individually for use in second mating.
5. Day 7: Vials from day 1 were scored for presence/absence of larvae and only females who produced larvae from the first mating were retained for use in remating trials.
6. Day 9: Previously mated females were transferred without anesthesia to an isolated virgin male’s vial and mating behavior (copulation latency and duration) observed for 3 hours. After assessing remating, all flies were discarded. Progeny from the second mating were not scored because the paternity of these progeny cannot be determined based on phenotypic traits, and therefore cannot be used to confirm whether a second mating successfully occurred.

## Statistical analysis

For the comparison of remating rates between allopatric and sympatric females that had initially mated with heterospecific males we used parametric survival regression because the data violated the proportional hazards assumption (survival curves crossed over the entire time interval); in this case we used parametric survival regression with an exponential distribution (determined from examining model/Weibull assumptions; Kleinbaum and Klein 2005). Note that our qualitative findings did not vary based on the specific statistical model used (data not shown), although in general proportional hazards models make fewer assumptions about the data (they are semi-parametric).

We included female genotype in the proportional hazard models to account for correlated observations within a given female genotype. This approach utilizes generalized estimating equations (GEE) and is similar to a random effect in a mixed effect model (Therneau and Grambsch 2000). This term was not included in the parametric survival regression as it is not compatible with the exponential survival regression.

## Multiple correcting

In our analysis we rely on two main data sets: first mating and remating data. Because we carry out several complementary tests using these data we corrected for multiple testing using the Benjamini-Hochberg method (Benjamini and Hochberg 1995). The general principle of this

method is that p-values are ranked and then the largest value of i (k) for which

𝑘

𝑃(𝑘) ≤𝑞 𝑚

is found where m is the total number of tests and q is the false discovery rate for which you want to control. We carried out this correction for both the first mating (Supplemental Table 1) and remating (Supplemental Table 2) data. After this correction all P-values that were initially <0.05 remained significant for first mating, but two comparisons were no longer significant for remating tests (see main text for these cases).

# Supporting Results

## Copulation latency and duration

In all first and second matings in all crosses, copulation duration ranged between 1 and 7 minutes in length. Supplementary Table 3 shows data for the sample size, mean, and standard deviation for copulation latency and duration, for all matings that were directly observed within the 3 hour observation period. Note that, because of the small sample sizes we have here for observed heterospecific copulations in the first 3 hours, the reported +/- SD here is likely to be quite inflated for both copulation latency and duration.

## Excluding possible date effects on our results for heterospecific first matings

In our experiment, a second stage of heterospecific pairings was performed after a first complete stage of both conspecific and heterospecific matings (which were run in parallel), specifically to increase our replication of assays involving heterospecific first matings. (As the results show, the frequency of heterospecific first matings was much lower than the frequency of conspecific first matings; main text.) To exclude the possibility that our results from the first and second stage of the experiment might differ due to date effects, we analyzed the differences in first mating rates between heterospecific matings in the initial full block and the heterospecific-only second stage and found no significant difference between the two for allopatric or sympatric matings (Allopatric: χ2 =0.0302; *P* = 0.8621; Sympatric: χ2 =1.1776; *P* = 0.2778). This indicates that date did not influence patterns of mating propensity in these female types, between the two stages.

## Survival curves for remating data

To evaluate whether *D. pseudoobscura* females differed in their readiness to remate depending on the identity of the first male they mated with, we compared the frequency of remating and the copulation latency in remating trials following three classes of first mating: with conspecific males from their own population, with conspecific males from a different population, or with heterospecific males. In the main text we report results with isofemale lines combined, but our observations for individual isofemale lines (Supplemental Figure 1) are consistent with our inferences from the more general analyses. As Supp. Figure 1 shows, there are differences in the specific progression of remating for each female line, following first matings with males of each type. However, most (5/6) of female lines show a consistent pattern in which control (‘own’ first mating) lines are equally or more rapid in the rate of remating, and the final proportion of remating after 3 hours, compared to the other two classes of first mating—especially first matings with Heterospecific males; this is consistent with our statistical inferences across all data (in the main text). Observed curves for ‘Diff’ and ‘Het’ matings were somewhat more idiosyncratic, consistent with our inability to distinguish them statistically (main text). Regardless, overall these line-specific analyses indicate that our broader inference—that females are most able to resist suppression of remating after mating with own males—is recapitulated in line-specific observations. The one female line (MSH3) for which we could not clearly distinguish these differences appeared to be equally unable to resist remating suppression, regardless of first male identity.

# References

Kleinbaum, D. G., and M. Klein, 2005 *Survival analysis: a self-learning text*. Springer, New York.

Noor, M. A. F., 1997 Genetics of sexual isolation and courtship dysfunction in male hybrids of *Drosophila pseudoobscura* and *Drosophila persimilis*. Evolution 51: 809-815.

Therneau, T. M, P. M. Grambsch, 2000 *Modeling Survival Data: Extending the Cox Model*. Statistics for

Biology and Health.


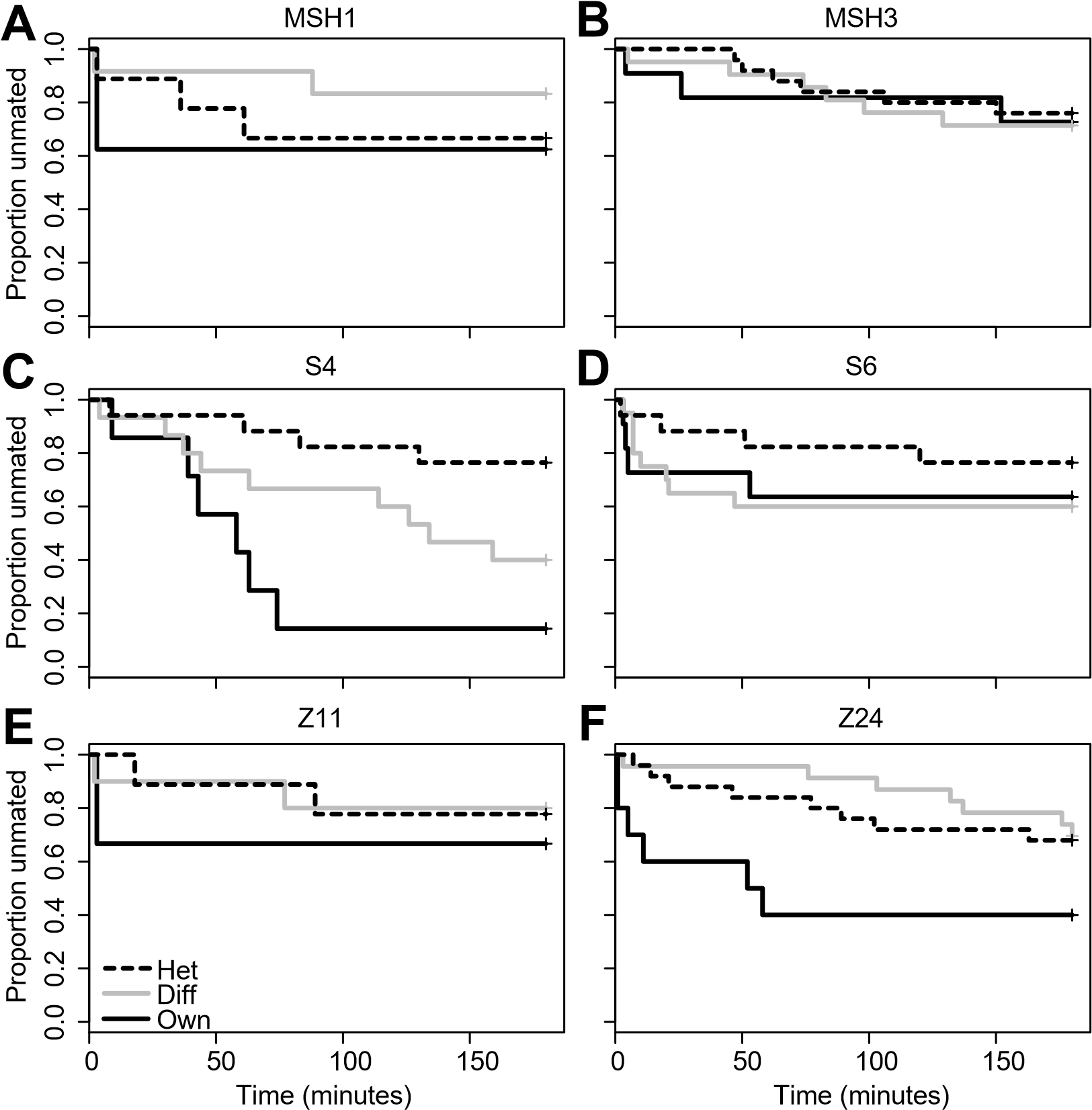


**Figure S1:** Survival curves showing remating latencies for each of the six examined isofemale lines when mated to different classes of first males: Own (conspecific male from the same population as the female); Diff (conspecific male from a different population); Hetero (heterospecific male). MSH= Mt St Helena population (sympatric); S=Sierra population

(sympatric); Z=Zion population (allopatric).

**Table S1.** Multiple correction for first mating data following the Benjamini-Hochberg procedure. Bold rows indicate significant p-values after correction

| Original Ranked p-values | P(k) from Benjamini-Hochberg |
| --- | --- |
| **2.2e-16** | **0.005** |
| **8.3e-10** | **0.010** |
| **7.1e-5** | **0.015** |
| **0.007** | **0.020** |
| **0.009** | **0.025** |
| 0.623 | 0.030 |
| 0.671 | 0.035 |
| 0.722 | 0.040 |
| 0.8486 | 0.045 |
| 0.994 | 0.050 |

**Table S2**. Multiple correction for remating data following the Benjamini-Hochberg procedure. Bold rows indicate significant p-values after correction. * indicates no longer significant after correcting for multiple testing.

| Original Ranked p-values | P(k) from Benjamini-Hochberg |
| --- | --- |
| **0.0005** | **0.0055** |
| **0.0055** | **0.0111** |
| 0.0219* | 0.0166 |
| 0.0352* | 0.0222 |
| 0.1047 | 0.0277 |
| 0.5280 | 0.0333 |
| 0.5447 | 0.0388 |
| 0.5840 | 0.0444 |
| 0.6569 | 0.0500 |

**Table S3**. Sample size, mean, and standard deviation for copulation latency for all copulations (in minutes) directly observed within the initial 3 hour observation period.

| **Cross** | **Female** | **First Cross Male** |  | **First mating** | | **Second Mating** | | |
| --- | --- | --- | --- | --- | --- | --- | --- | --- |
|  | (Sympatric) |  | *N* | *Mean* | *SD* | *N* | *Mean* | *SD* |
| Own | Sierra *D.pse* | Sierra *D.pse* | 19 | 4.16 | 10.32 | 12 | 30.66 | 27.17 |
| Diff | Sierra *D.pse* | Mt. St. Helena *D.pse* | 16 | 2.06 | 2.57 | 9 | 44.3 | 45.05 |
| Diff | Sierra *D.pse* | Zion *D.pse* | 18 | 1.22 | 1.35 | 5 | 55.71 | 63.22 |
| Het | Sierra *D.pse* | Sierra *D.per* | 4 | 5 | 1.41 | 11 | 11.64 | 22.13 |
| Het | Sierra *D.pse* | Mt. St. Helena *D.per* | 3 | 11 | 3.61 | 11 | 36 | 49.99 |
|  |  |  |  |  |  |  |  |  |
|  | (Sympatric) |  |  |  |  |  |  |  |
| Diff | Mt. St. Helena *D.pse* | Sierra *D.pse* | 18 | 1.5 | 2.62 | 5 | 76.8 | 47.75 |
| Own | Mt. St. Helena *D.pse* | Mt. St. Helena *D.pse* | 17 | 1.69 | 2.80 | 7 | 31.83 | 59.57 |
| Diff | Mt. St. Helena *D.pse* | Zion *D.pse* | 17 | 0.94 | 0.97 | 10 | 66.2 | 78.05 |
| Het | Mt. St. Helena *D.pse* | Sierra *D.per* | 3 | 7.33 | 3.21 | 7 | 75.86 | 39.96 |
| Het | Mt. St. Helena *D.pse* | Mt. St. Helena *D.per* | 1 | 6 | 0 | 8 | 11.88 | 19.60 |
|  |  |  |  |  |  |  |  |  |
|  | (Allopatric) |  |  |  |  |  |  |  |
| Diff | Zion *D.pse* | Sierra *D.pse* | 18 | 1.06 | 1.03 | 5 | 112.8 | 42.12 |
| Diff | Zion *D.pse* | Mt. St. Helena *D.pse* | 15 | 0.67 | 0.62 | 5 | 105.2 | 96.67 |
| Own | Zion *D.pse* | Zion *D.pse* | 15 | 2.4 | 3.91 | 8 | 39.88 | 64.20 |
| Het | Zion *D.pse* | Sierra *D.per* | 2 | 3 | 4.24 | 10 | 26.7 | 33.00 |
| Het | Zion *D.pse* | Mt. St. Helena *D.per* | 3 | 11 | 3.61 | 11 | 36 | 49.99 |
|  |  |  |  |  |  |  |  |  |

**Table S4**. Sample size, mean, and standard deviation for copulation duration for all copulations (in minutes) directly observed within the initial 3 hour observation period.

| **Cross** | **Female** | **First Cross Male** |  | **First mating** | | **Second Mating** | | |
| --- | --- | --- | --- | --- | --- | --- | --- | --- |
|  | (Sympatric) |  | *N* | *Mean* | *SD* | *N* | *Mean* | *SD* |
| Own | Sierra *D.pse* | Sierra *D.pse* | 19 | 3.32 | 1.25 | 12 | 3.17 | 1.27 |
| Diff | Sierra *D.pse* | Mt. St. Helena *D.pse* | 16 | 3.38 | 1.41 | 9 | 4.70 | 1.34 |
| Diff | Sierra *D.pse* | Zion *D.pse* | 18 | 3.61 | 1.03 | 5 | 3.42 | 0.98 |
| Het | Sierra *D.pse* | Sierra *D.per* | 4 | 4.00 | 0.82 | 11 | 3.18 | 1.60 |
| Het | Sierra *D.pse* | Mt. St. Helena *D.per* | 3 | 2.67 | 0.58 | 11 | 3.36 | 1.03 |
|  |  |  |  |  |  |  |  |  |
|  | (Sympatric) |  |  |  |  |  |  |  |
| Diff | Mt. St. Helena *D.pse* | Sierra *D.pse* | 18 | 3.79 | 0.89 | 5 | 3.40 | 0.55 |
| Own | Mt. St. Helena *D.pse* | Mt. St. Helena *D.pse* | 17 | 4.31 | 1.25 | 7 | 3.67 | 1.37 |
| Diff | Mt. St. Helena *D.pse* | Zion *D.pse* | 17 | 3.53 | 1.12 | 10 | 3.20 | 1.10 |
| Het | Mt. St. Helena *D.pse* | Sierra *D.per* | 3 | 2.67 | 2.08 | 7 | 3.43 | 1.13 |
| Het | Mt. St. Helena *D.pse* | Mt. St. Helena *D.per* | 1 | 2 | 0 | 8 | 4.13 | 1.55 |
|  |  |  |  |  |  |  |  |  |
|  | (Allopatric) |  |  |  |  |  |  |  |
| Diff | Zion *D.pse* | Sierra *D.pse* | 18 | 3.12 | 1.05 | 5 | 3.2 | 2.17 |
| Diff | Zion *D.pse* | Mt. St. Helena *D.pse* | 15 | 3.73 | 0.88 | 5 | 3.8 | 0.84 |
| Own | Zion *D.pse* | Zion *D.pse* | 15 | 3.73 | 0.96 | 8 | 3.88 | 1.96 |
| Het | Zion *D.pse* | Sierra *D.per* | 2 | 5 | 0 | 10 | 2.8 | 1.03 |
| Het | Zion *D.pse* | Mt. St. Helena *D.per* | 3 | 2.67 | 0.58 | 11 | 3.36 | 1.03 |
